# Supplementary material for: Cryo-EM structure of 5-HT3A receptor in its resting conformation
Source: Nat Commun. 2018 Feb 6;9:514. doi: 10.1038/s41467-018-02997-4 (PMC5802770; doi:10.1038/s41467-018-02997-4)
Supplement: Supplementary file 1 — Supplementary Information [file 41467_2018_2997_MOESM1_ESM.docx]

**
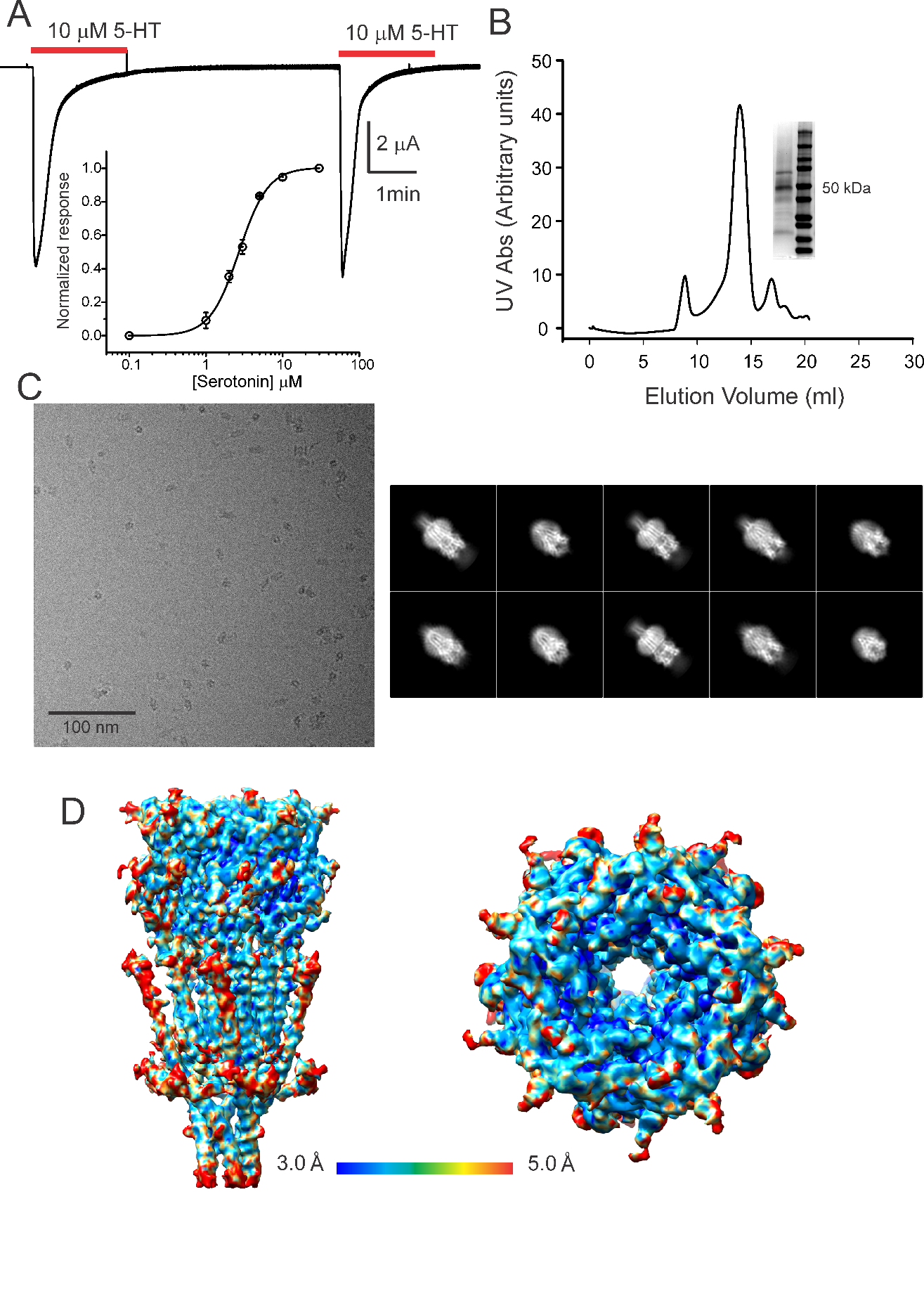
Supplementary Figure 1. Full-length 5-HT_3A_R functional and structural characterization.** (A) Two-electrode voltage clamp recording of 5-HT_3A_R expressed in oocytes. Currents were elicited in response to application of 10 μM serotonin (5-HT) with a holding membrane potential of -60 mV. Robust inward currents are observed that desensitize in the presence of serotonin. The ligand-pulses were interspaced by perfusion with the ND96 solution to fully recover the channels. The dose-response plot was generated by normalizing the peak current amplitude to that measured at 30 μM. The curve is a fit to the Hill equation yielding an EC_50_ of 2.7 + 0.09 μM and n_H_ of 2.3 + 0.17 for n = 3 The error bars are standard deviations. (B) Gel-filtration profile of 5-HT_3A_R expressed and purified from Sf9 cells. The main peak corresponds to the pentameric population which on the SDS-PAGE gel appears as a band at 50 kDa (C) A representative micrograph (out of 3550 micrographs) of 5-HT_3A_R in vitreous ice (Left). Selected 2D classes showing various orientations (Right). (D) Side view and top view of the 3D density map colored coded by the local resolution determined using the ResMap program^1^

**
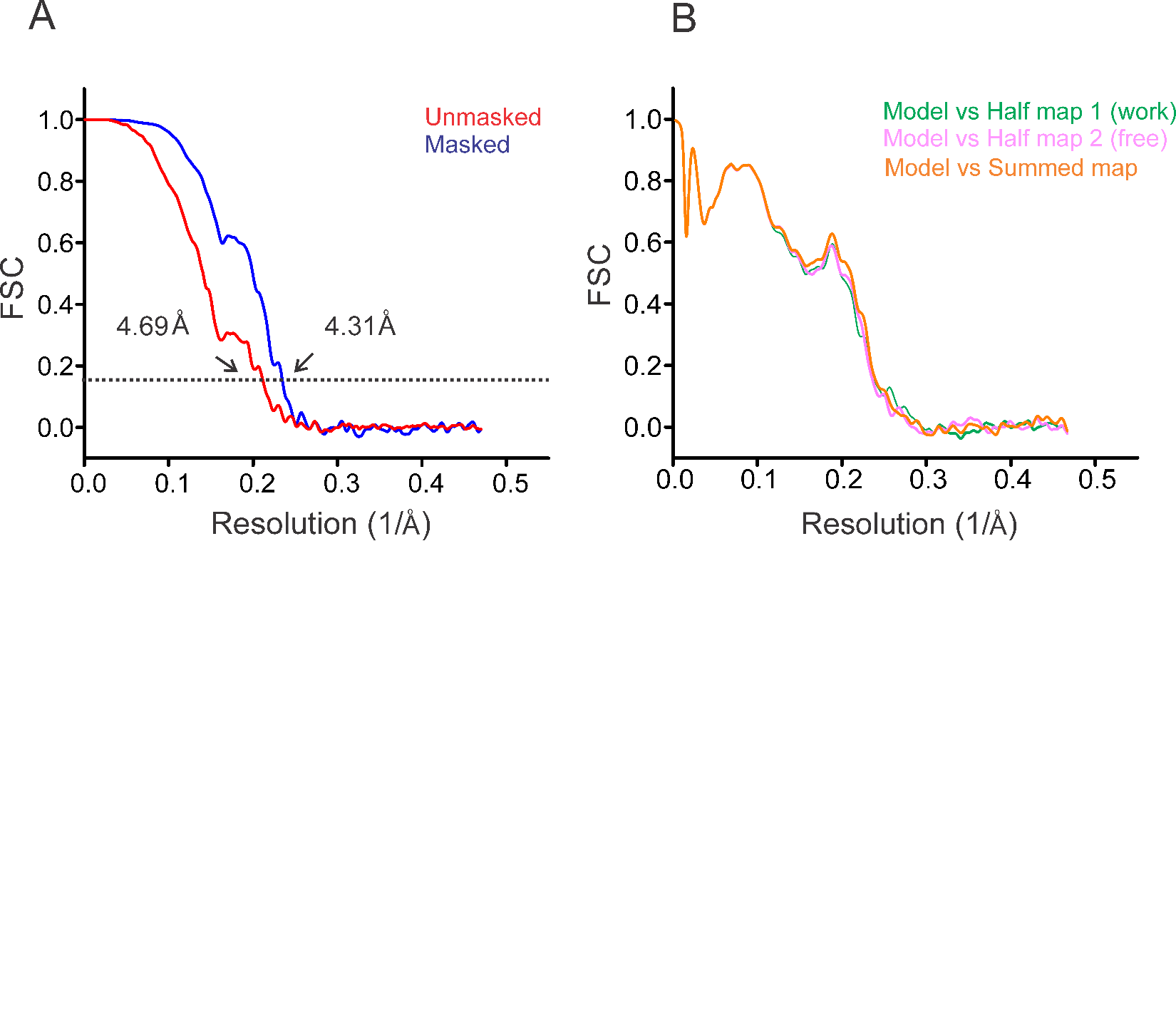
**

**Supplementary Figure 2. Resolution estimation and model validation.** (A) Fourier shell correlation (FSC) curves before (unmasked, red) and after (masked, blue) post-processing in RELION. The dashed line represents an FSC of 0.143. (B) For cross validation, FSC curves of the refined model versus summed map (full dataset, orange), refined model versus half map 1 (used during refinement, green), and refined model versus half map 2 (not used during refinement, pink) are calculated.

**
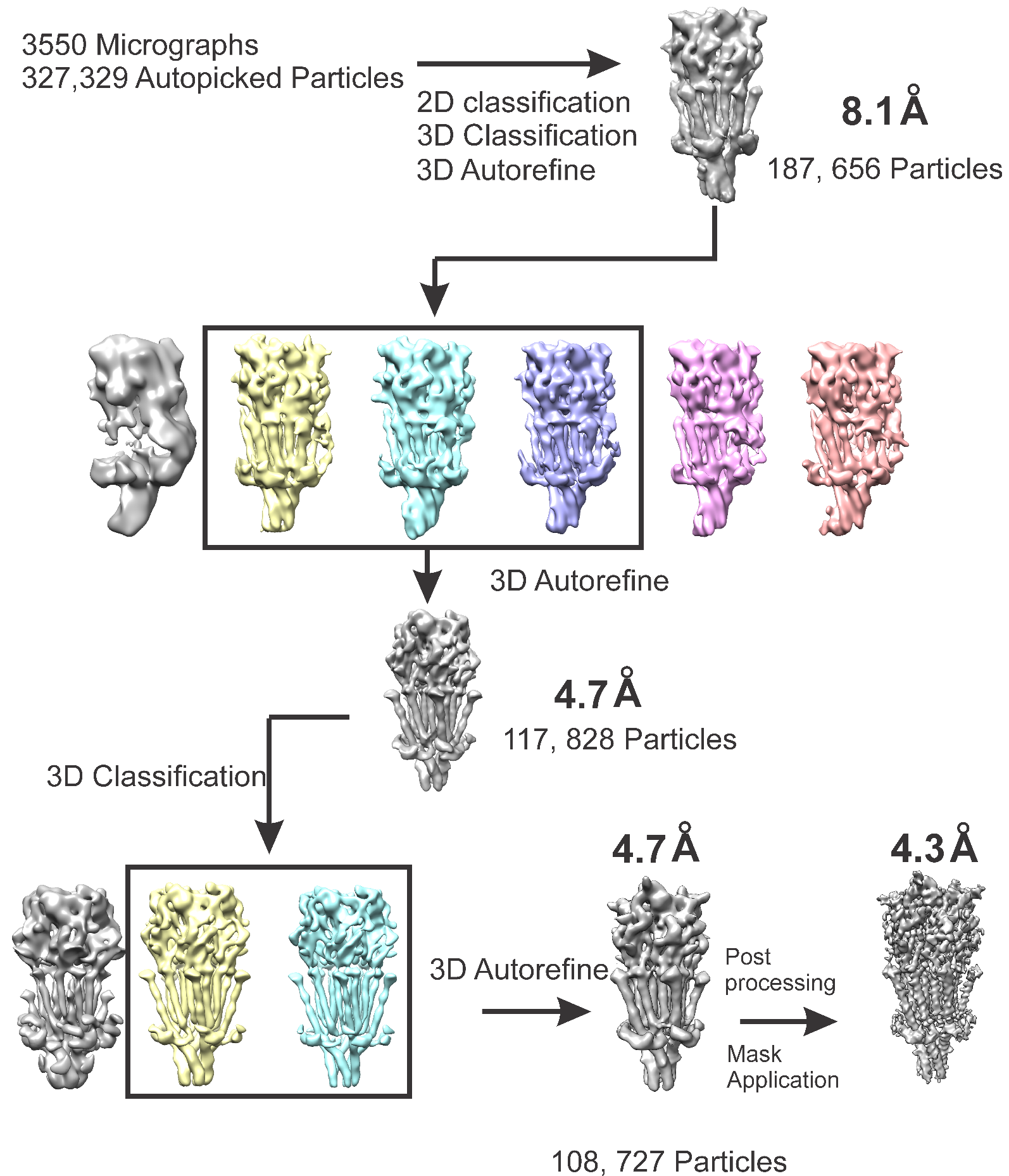
**

**Supplementary Figure 3**. **Data Processing workflow**. A schematic depiction of the various steps in the data processing that led to a 4.3 Å reconstruction. Classes with boxed regions were used for further analysis.

**
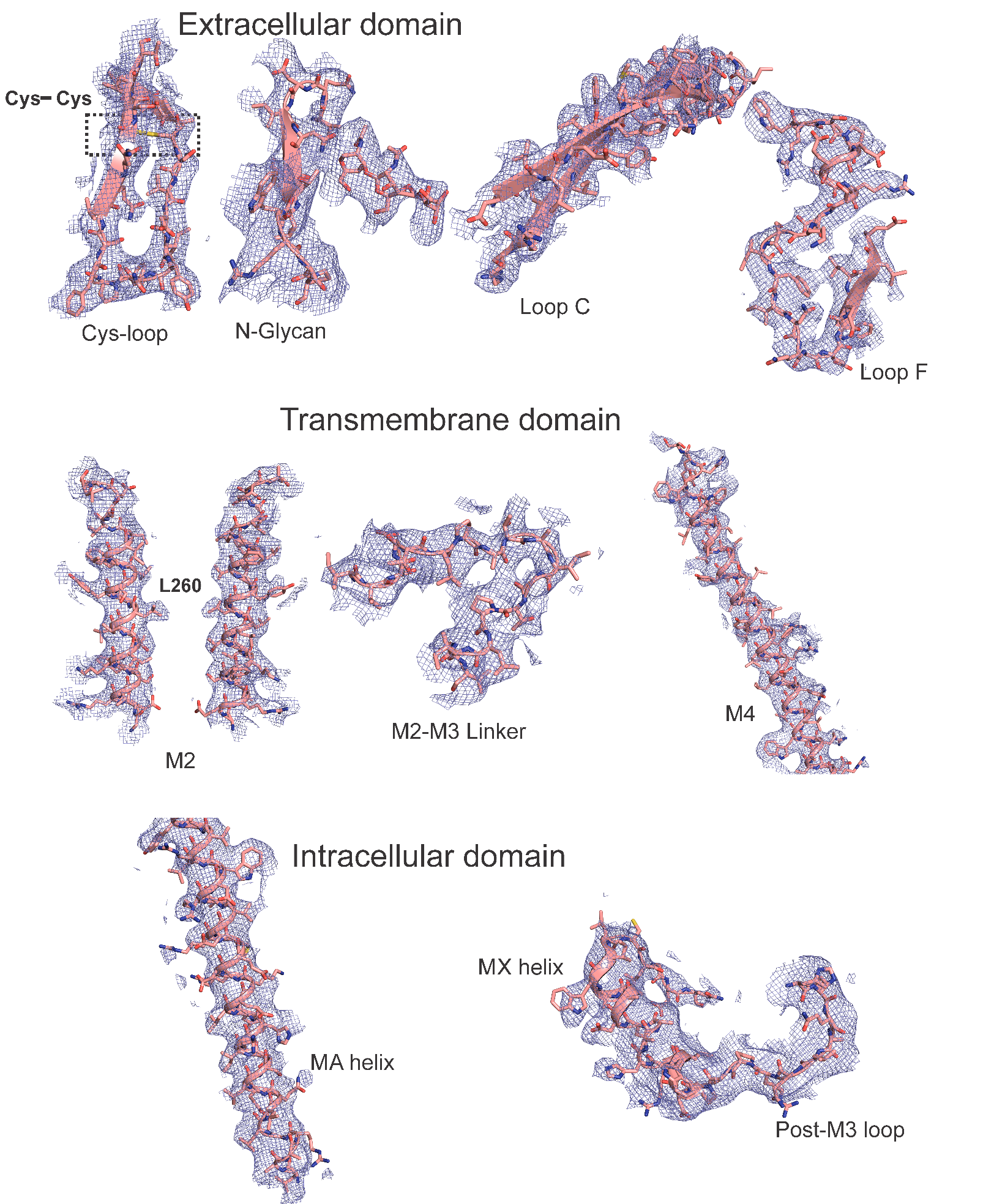
**

**Supplementary Figure 4. Validation of the 5-HT_3A_R structural model.** (A) Sections of density map (mesh) from the final reconstruction is overlaid with the 5-HT_3A_R model (shown as a cartoon) for various regions of the channel. Several representative residues are depicted as sticks.

**
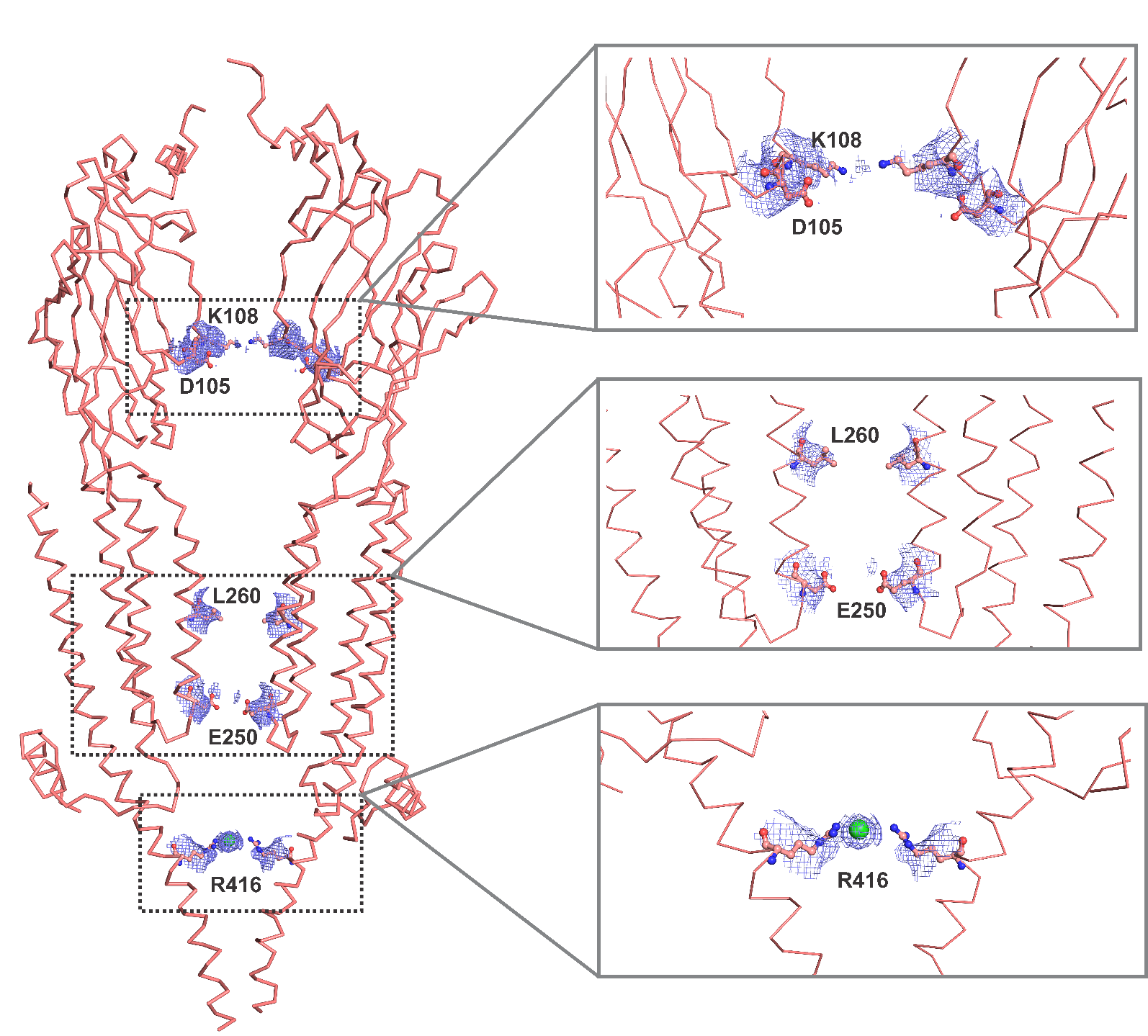
**

**Supplementary Figure 5. Validation of constriction sites.** Sections of electron density map (mesh) around the residues in the ECD, TMD, and ICD that appear as constriction points in the ion conduction pathway.

**
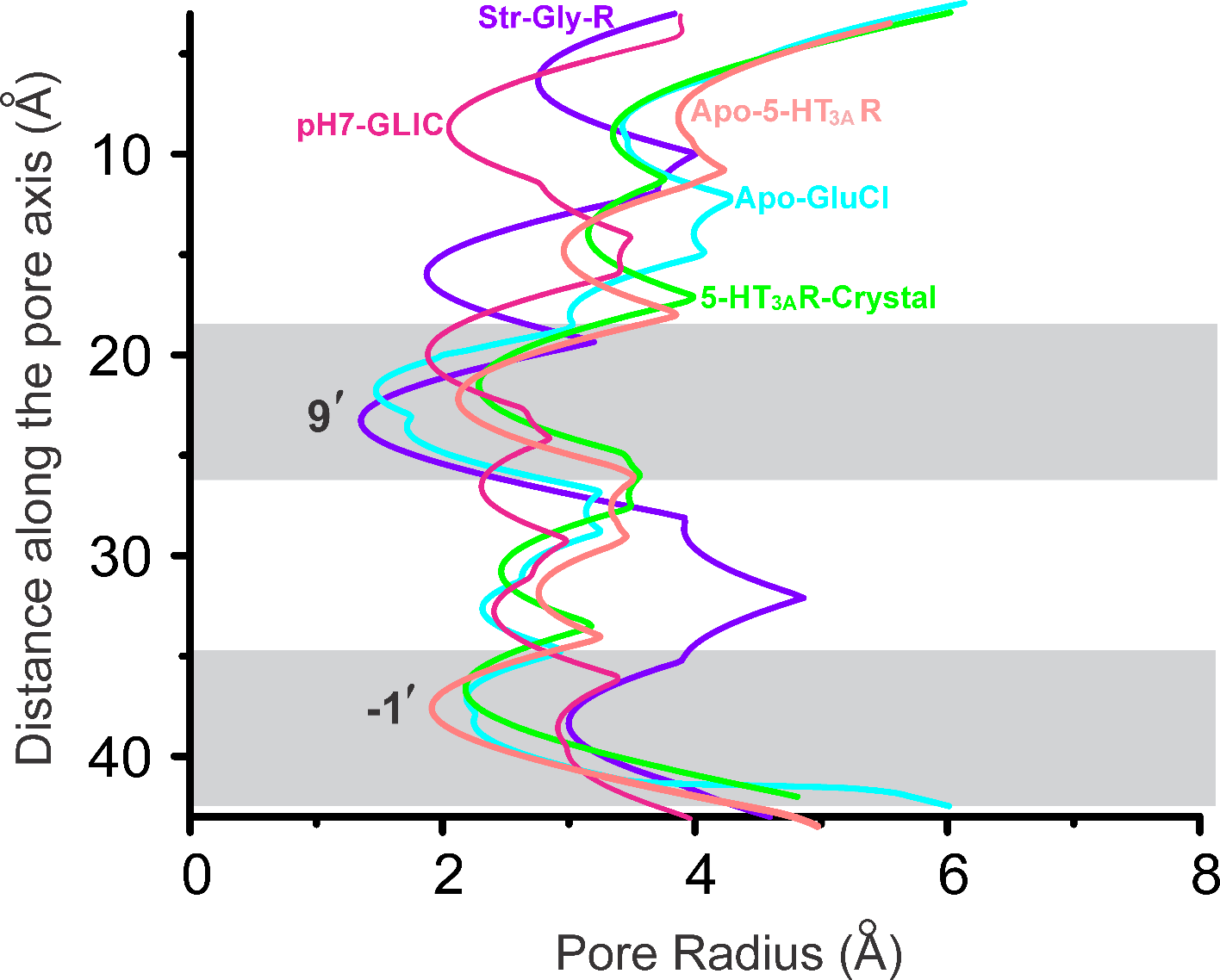
**

**Supplementary Figure 6. Pore Profile of pLGICs in non-conductive conformations.** The pore profile generated by the HOLE program for various pLGICs (PDB-IDs: 5-HT_3A_-R crystal structure: 4PIR^2^; strychnine-GlyR:3JAD^3^; pH7-GLIC: 4NPQ^4^; and Apo-GluCl: 4TNV^5^ . The pore radii is plotted along the permeation pathway. The 9′ and -1′ positions are highlighted.

**
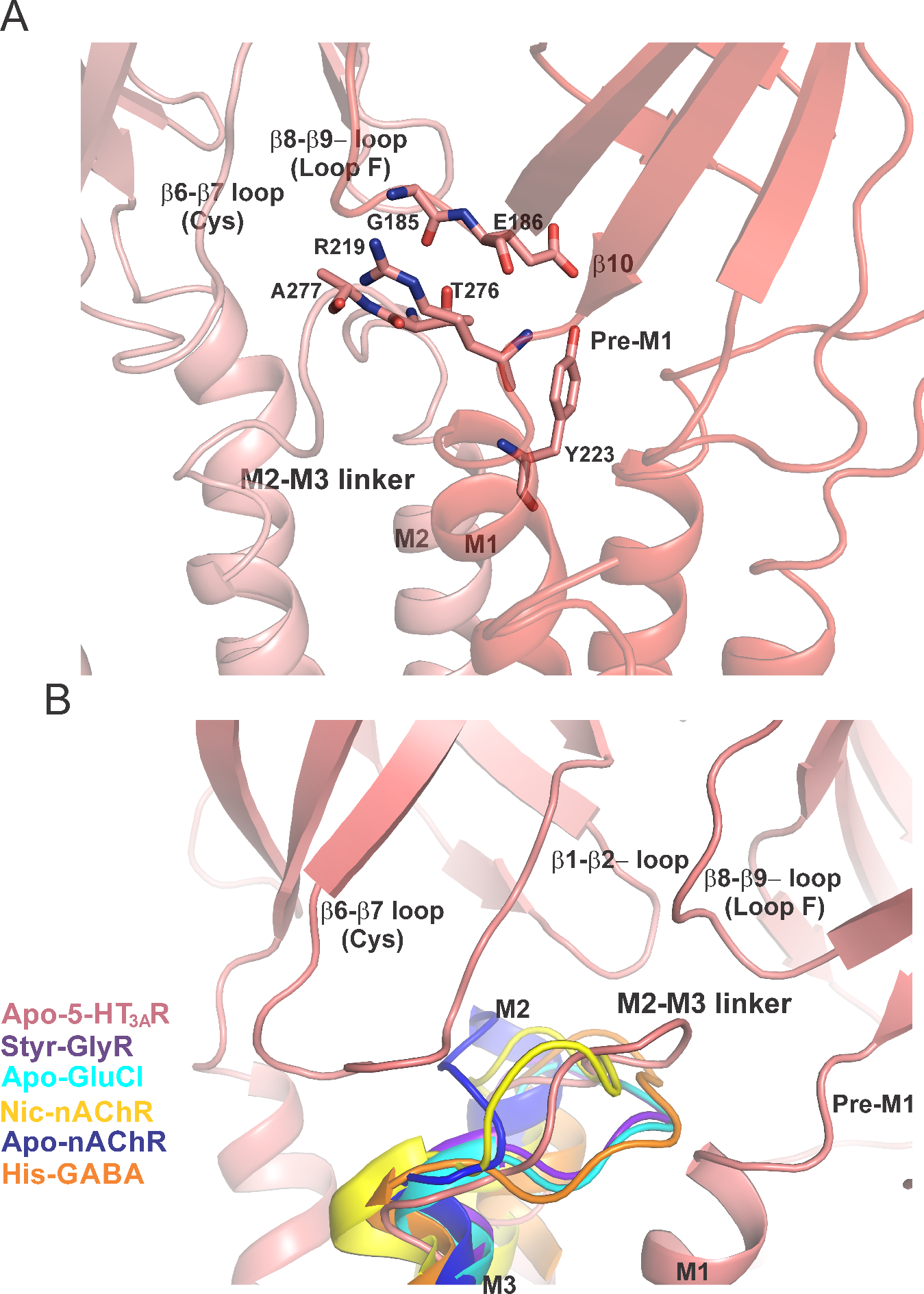
Supplementary Figure 7. Inter-subunit interaction at the ECD-TMD interface.** (A) The principal (+) subunit is shown in wheat and the complementary (-) subunit is shown in red. The M2-M3 linker from the (+) subunit interacts with the (-) subunit at the pre-M1 region (Ala277-Arg219) and at the β8-β9 linker (Thr276-Gly185). (B) A comparison of the position of M2-M3 linker as seen in various pLGIC structures (PDB-IDs: Styr-GlyR: 3JAD^3^; Apo-GluCl: 4TNV^5^; Nic-nAChR: 5KXI^6^; Apo-nAChR: 2BG9^7^; and His-GABA-β3: 4COF^8^). The principal subunit from these structures are superimposed on to apo-5-HT_3A_R.

**
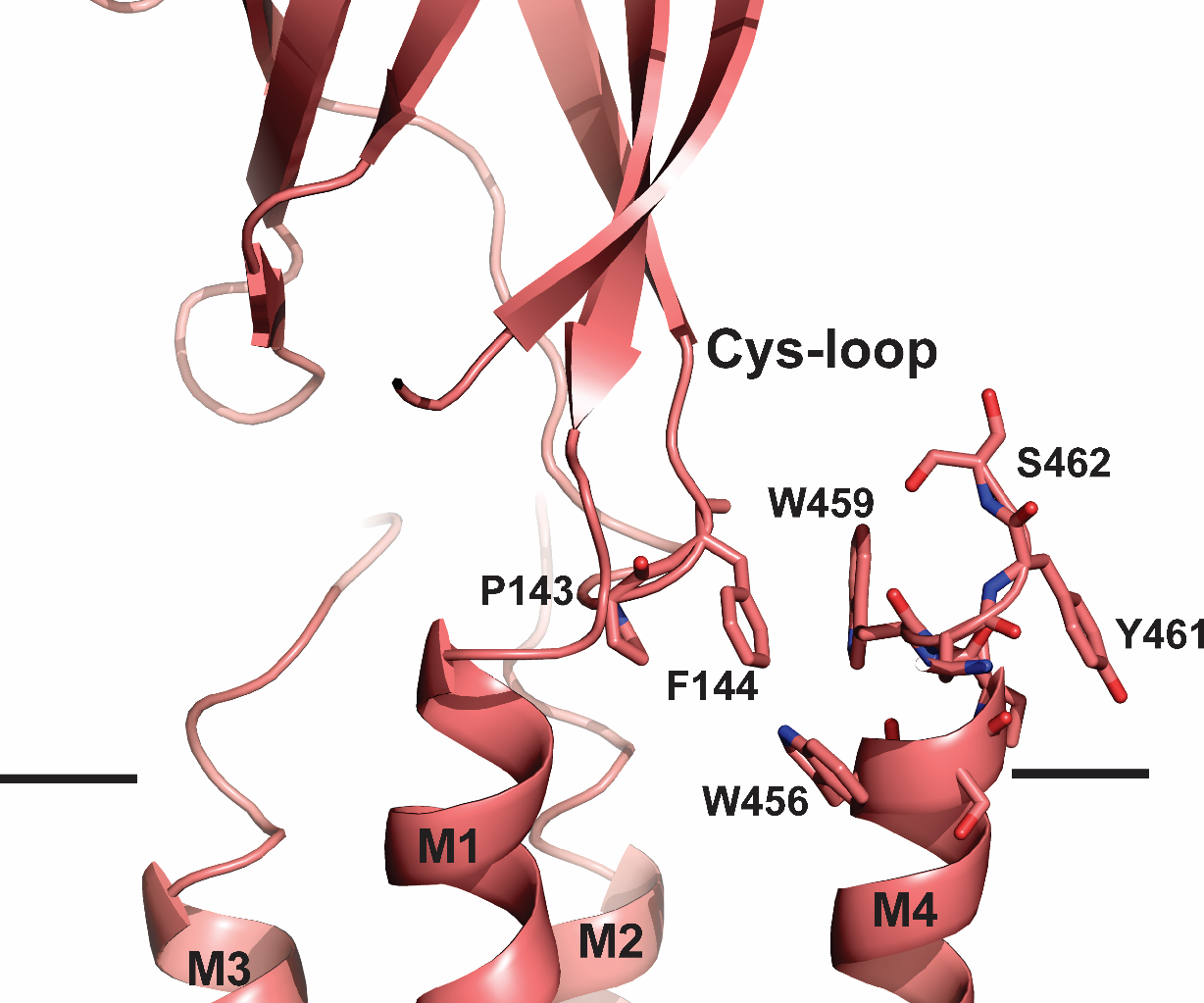
**

**Supplementary Figure 8. Inter-subunit interactions at the Cys-loop and M4.** Aromatic interaction between Cys-loop and the tip of M4. The solid line denotes putative membrane limits.

**
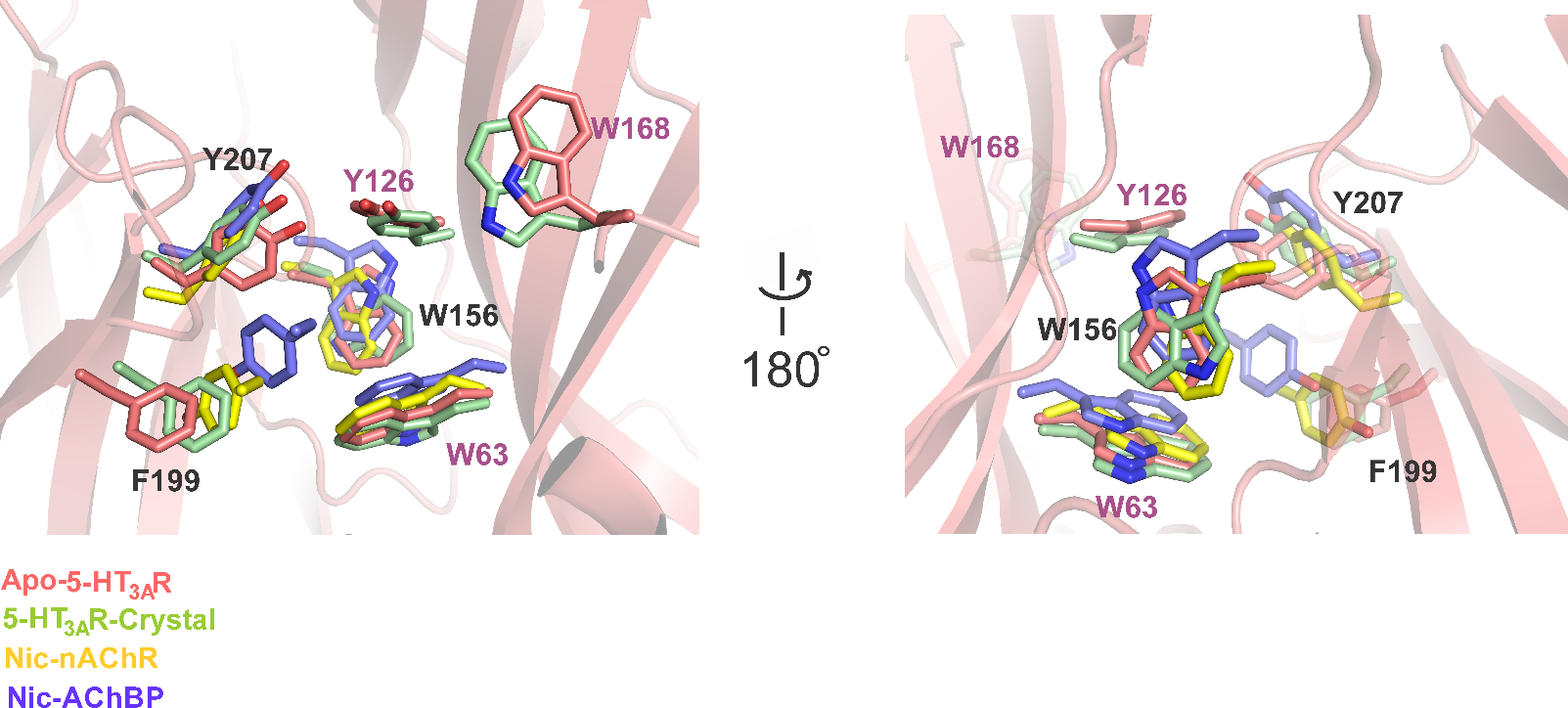
**

**Supplementary Figure 9. Comparison of ligand binding site.** Comparison of aromatic residues present at the binding site (PDB-IDs: Nic-nAChR: 5KXI^6^; Nic-AChBP: 1UW6^9^; 5-HT_3A_R crystal structure: 4PIR^2^).

**Supplementary References**

1 Kucukelbir, A., Sigworth, F. J. & Tagare, H. D. Quantifying the local resolution of cryo-EM density maps. *Nat Methods* **11**, 63-65 (2014).

2 Hassaine, G. *et al.* X-ray structure of the mouse serotonin 5-HT3 receptor. *Nature* **512**, 276-281 (2014).

3 Du, J., Lu, W., Wu, S., Cheng, Y. & Gouaux, E. Glycine receptor mechanism elucidated by electron cryo-microscopy. *Nature* **526** (7572), 224-229 (2015).

4 Sauguet, L. *et al.* Crystal structures of a pentameric ligand-gated ion channel provide a mechanism for activation. *Proc Natl Acad Sci U S A* **111**, 966-971 (2014).

5 Althoff, T., Hibbs, R. E., Banerjee, S. & Gouaux, E. X-ray structures of GluCl in apo states reveal a gating mechanism of Cys-loop receptors. *Nature* **512**, 333-337 (2014).

6 Morales-Perez, C. L., Noviello, C. M. & Hibbs, R. E. X-ray structure of the human alpha4beta2 nicotinic receptor. *Nature* **538**, 411-415 (2016).

7 Unwin, N. Refined structure of the nicotinic acetylcholine receptor at 4A resolution. *J Mol Biol* **346**, 967-989 (2005).

8 Miller, P. S. & Aricescu, A. R. Crystal structure of a human GABAA receptor. *Nature* **512**, 270-275 (2014).

9 Celie, P. H. *et al.* Nicotine and carbamylcholine binding to nicotinic acetylcholine receptors as studied in AChBP crystal structures. *Neuron* **41**, 907-914 (2004).
